# Supplementary material for: Influence of Graphene Oxide on Mechanical and Morphological Properties of Nafion® Membranes
Source: Nanomaterials (Basel). 2025 Jan 3;15(1):68. doi: 10.3390/nano15010068 (PMC11722737; doi:10.3390/nano15010068)
Supplement: Supplementary file 1 [file nanomaterials-15-00068-s001.zip › nanomaterials-3370649-supplementary.pdf]

Influence of Graphene Oxide on Mechanical and Morphological properties of Nafion® membranes

Carlos Ceballos-Alvarez, Maziar Jafari, Mohamed Siaj, Samaneh Shahgaldi and Ricardo Izquierdo

Supplementary Information

Figure S1

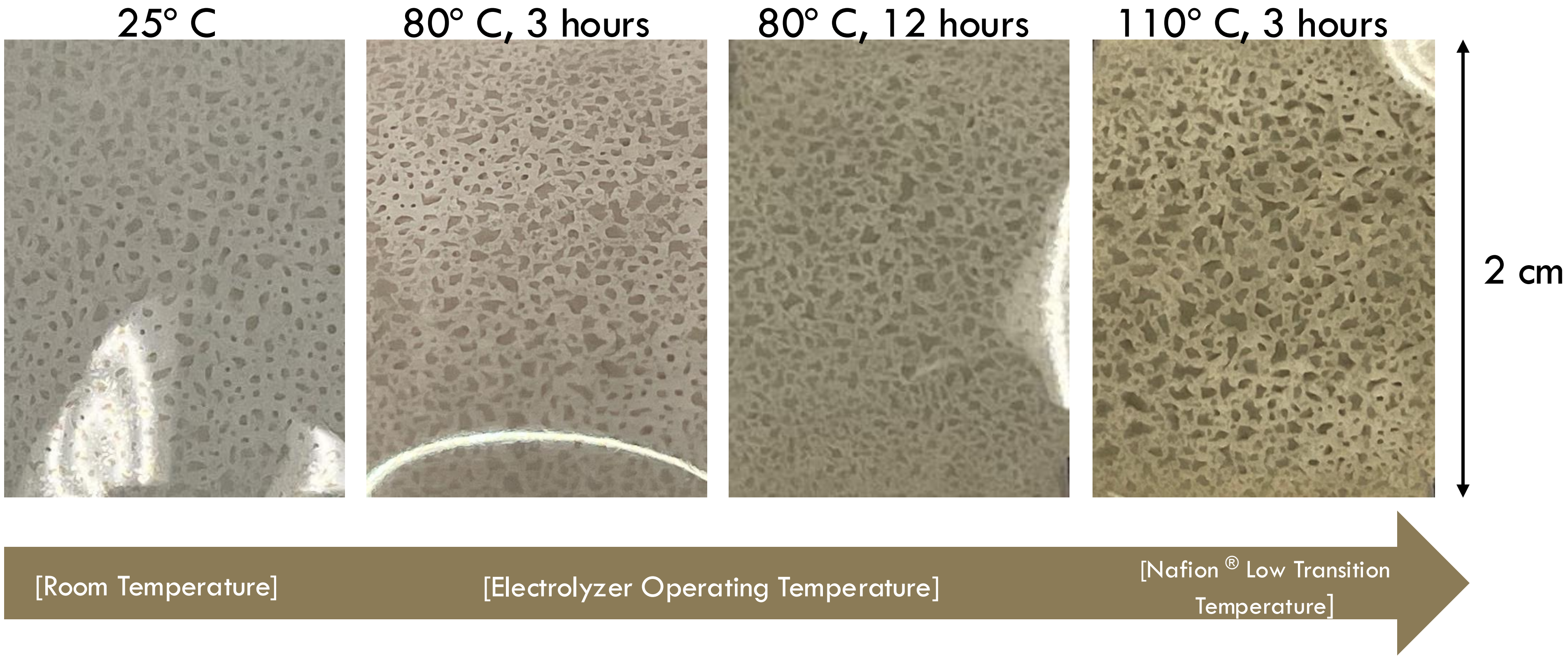

Optical image of the influence of thermal treatment of GO-Nafion® composite membranes

Figure S2

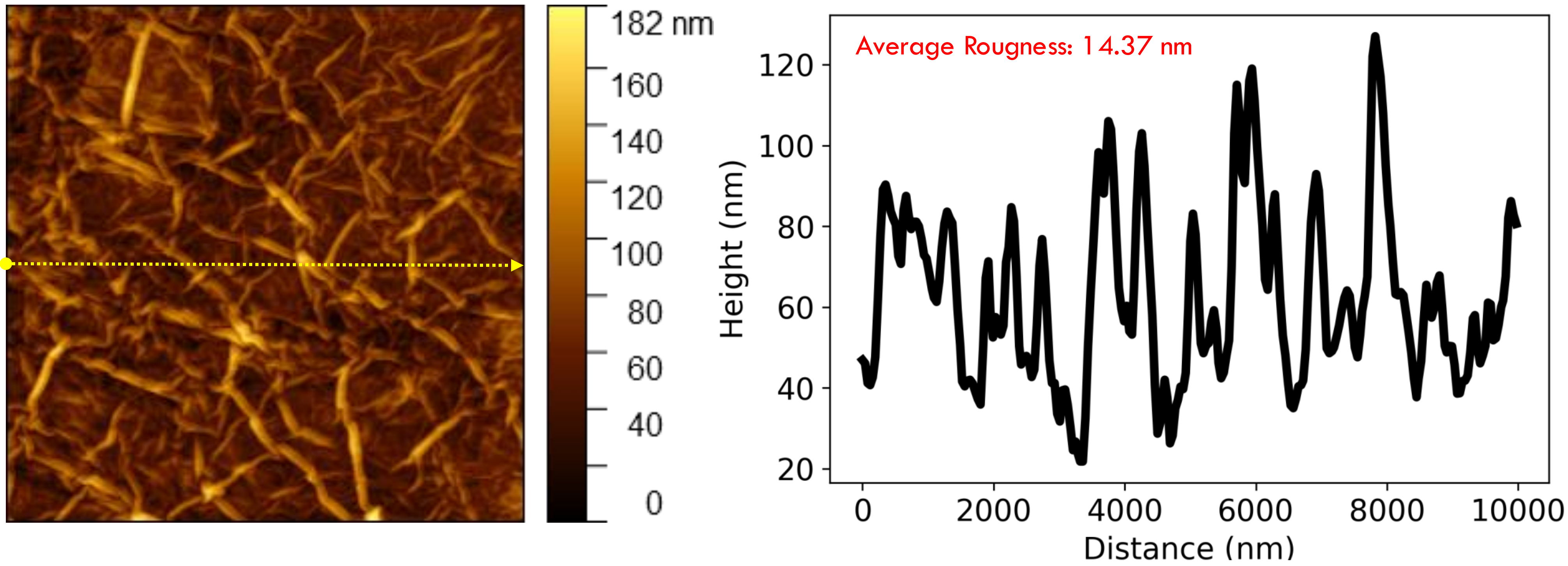

AFM Topography (left) and roughness (right) of the Graphene Oxide islands on the Nafion® 115 membrane

Figure S3

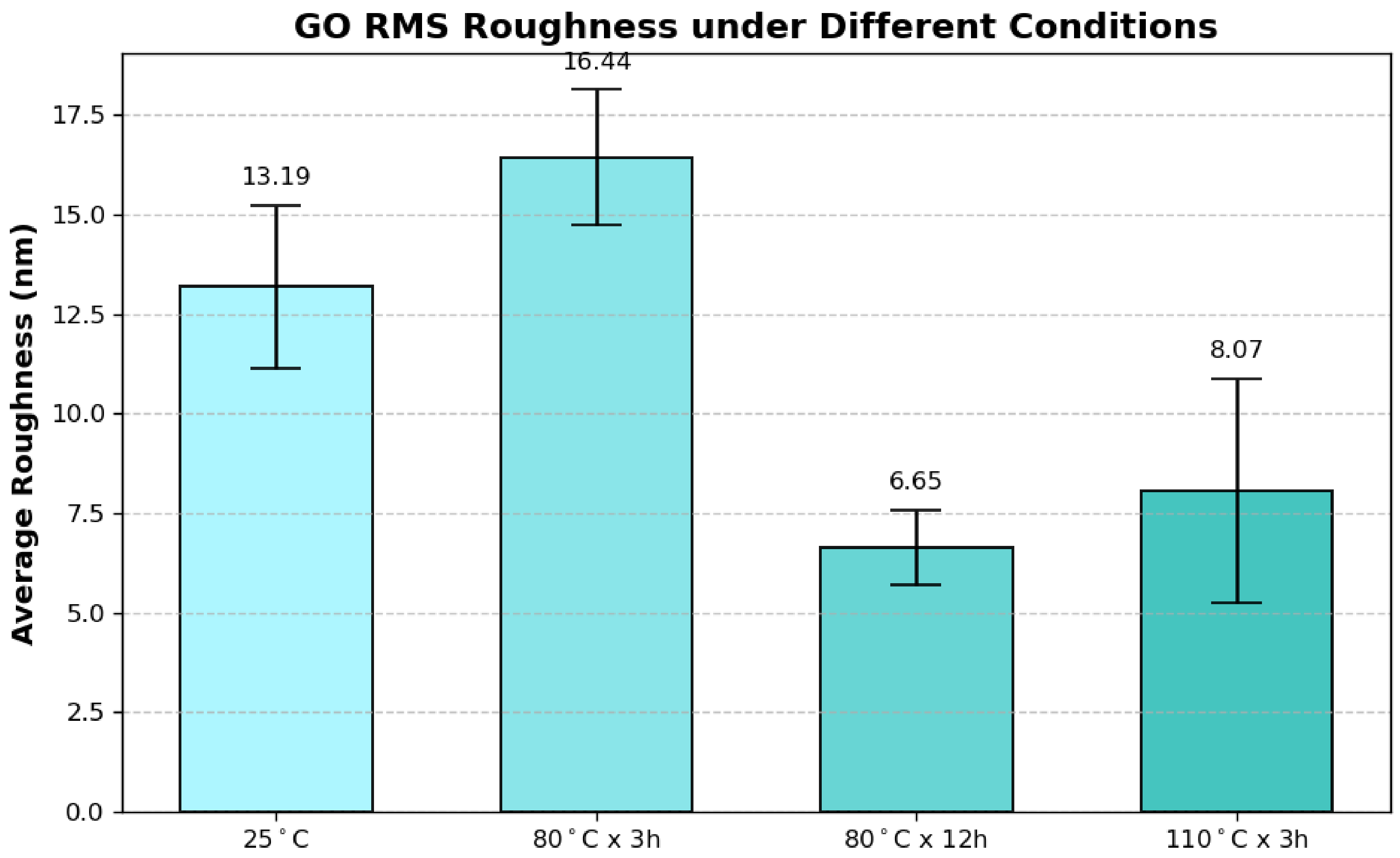

RMS roughness of the GO islands as a function of the different thermal treatments

Figure S4

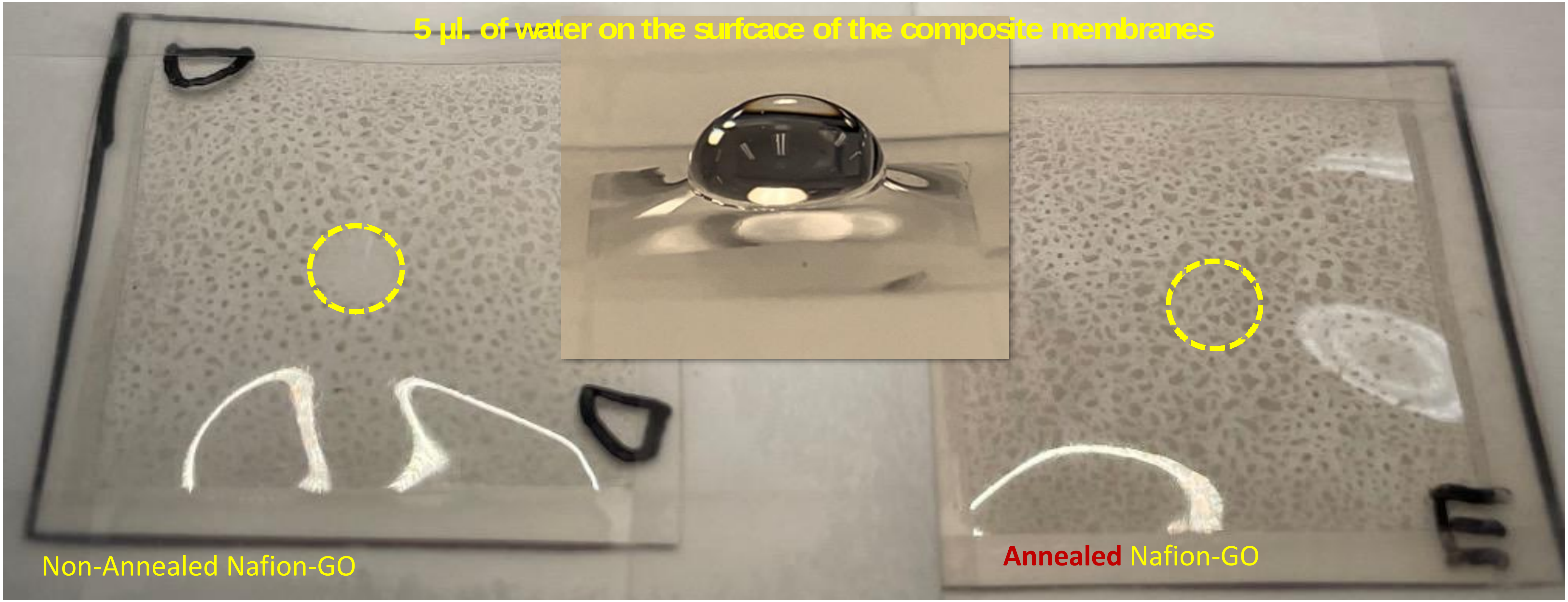

Photography of the non-annealed (left) and annealed (right) composite membranes after the contact angle test, the permanence of the GO layer can be observed only on the annealed sample.

Figure S5

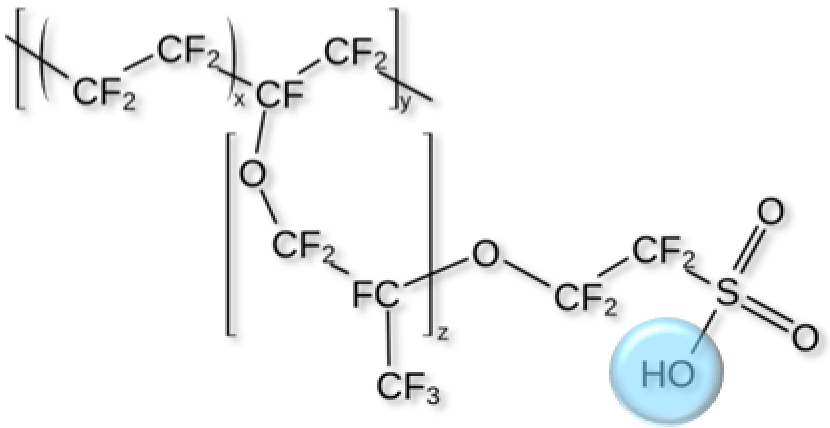

Nafion®

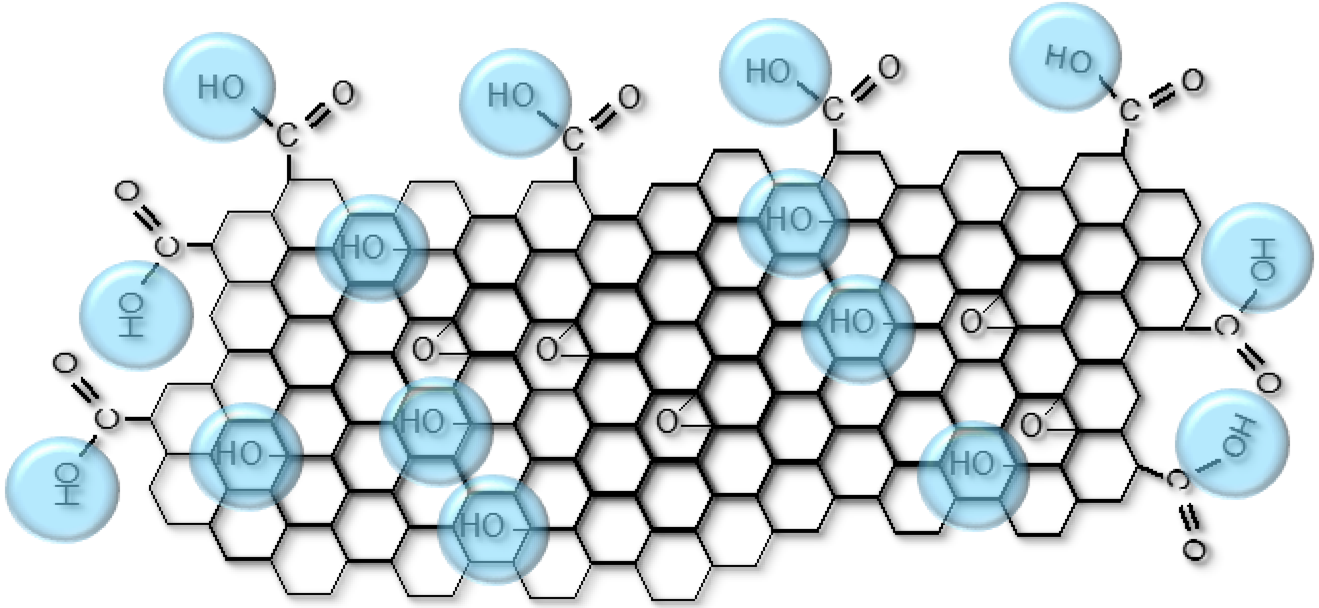

Graphene Oxide

Schematics of hydroxyl groups (blue zones) present on the chemical structures of Nafion® and graphene oxide.

Figure S6

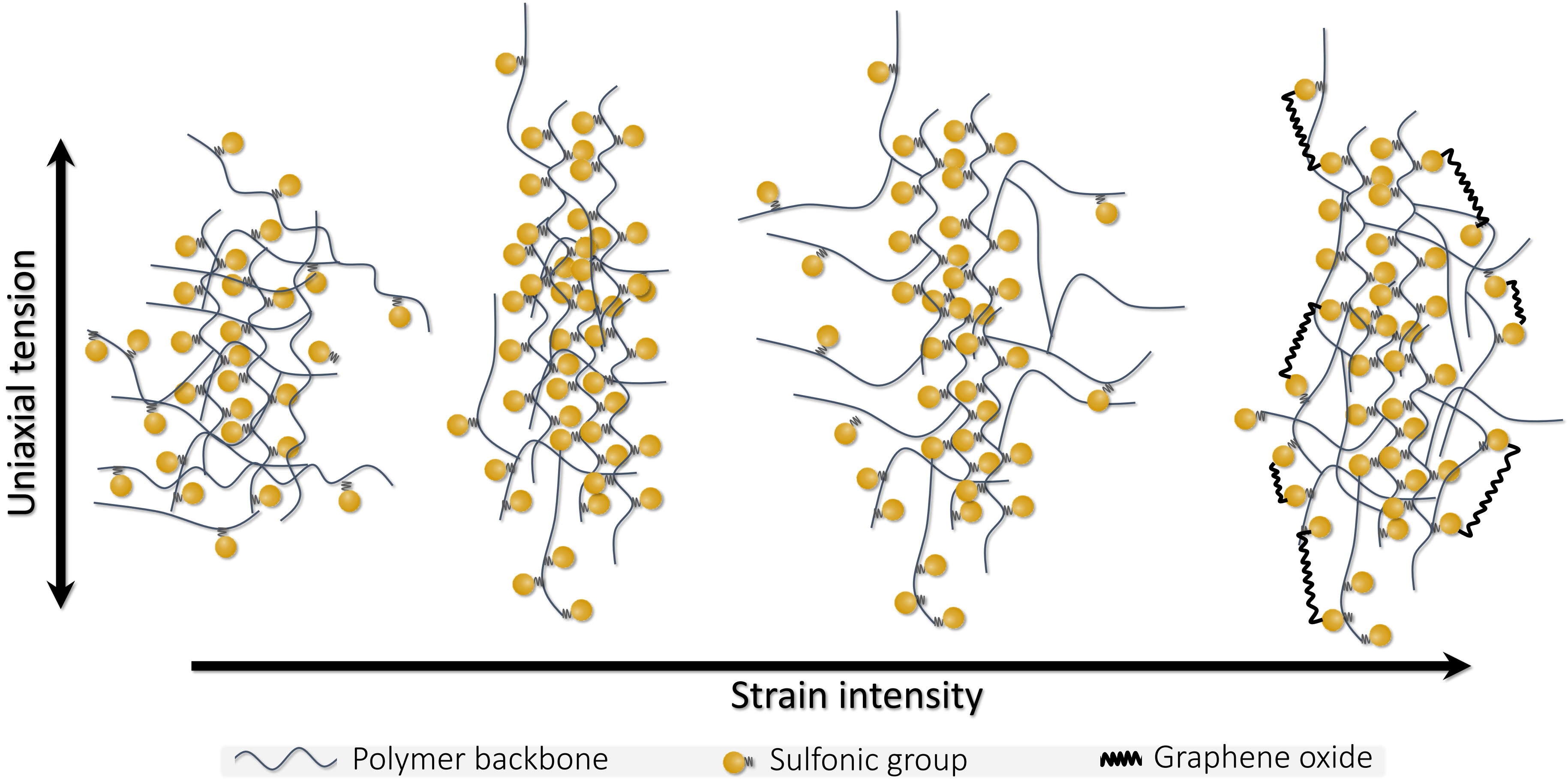

Conceptual model of how Nafion® deforms under uniaxial tension a) At small strain; b) At moderate strain; c) At high strain with some backbone chains breaking d) Hypothesis of GO staple effect absorbing part of the energy and improving the tensile strength in the polymer. Adapted from Silverstein, 2008 [1]

M. N. Silberstein, "Mechanics of Proton Exchange Membranes: Time, Temperature and Hydration dependence of the Stress-Strain behavior of Persulfonated Polytetrafluorethylene," Massachusetts Institute of Technology, 2008.

Figure S7

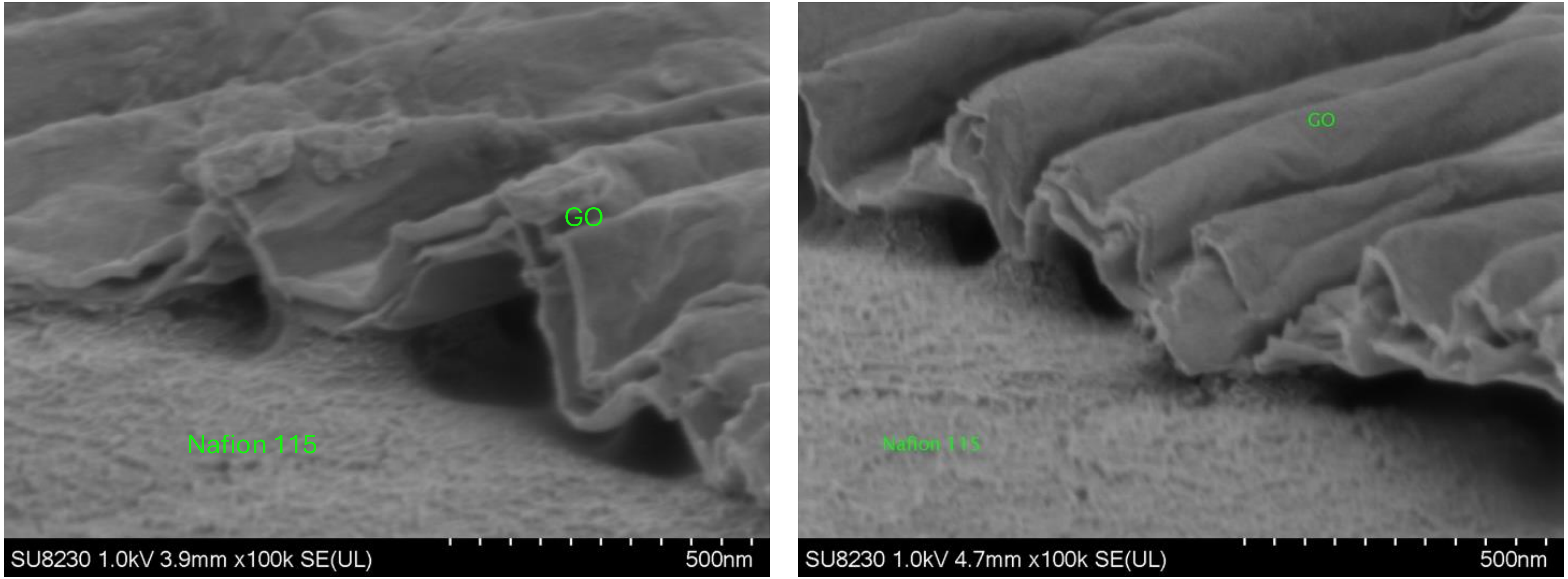

Scanning Electron Microscopy (SEM) images of multilayer graphene oxide deposited on the surface of a Nafion® 115 membrane. A two-layer configuration is visible on the left, while a three-layer configuration is observed on the right.

Figure S8

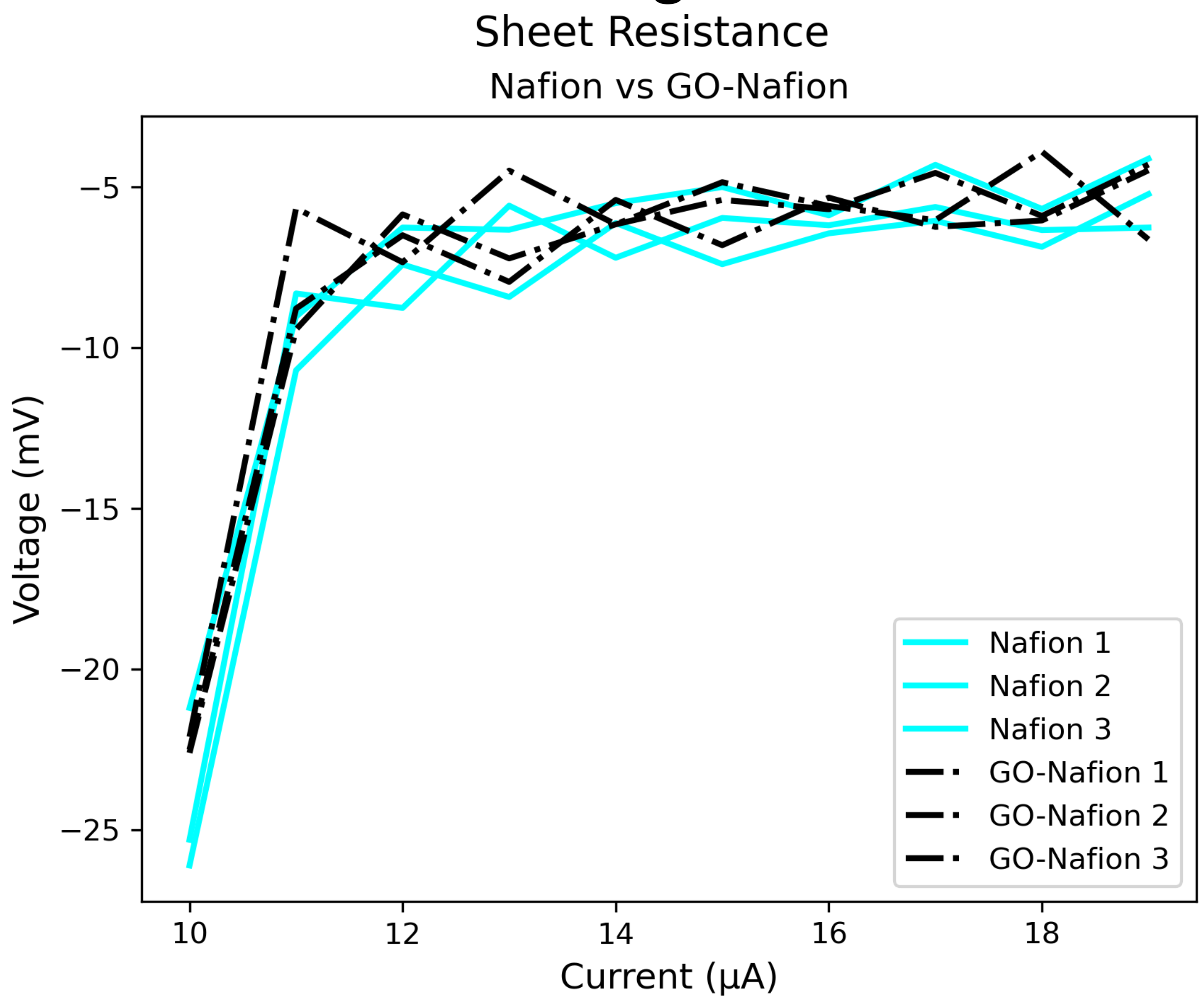

Sheet resistance of samples annealed at 80°C x 3 hours, showing Nafion samples (Cyan) and GO- Nafion® composite membranes (Dashed black)

Table S1

Table S1. Mechanical, hydrophilicity and electrical insulation comparison between nanocarbon reinforcement agents used in Nafion composites for PEMWE applications.

Composite Material	Mechanical Improvement*	Effect on Hydrophilicity*	Electrical Conductivity*	Reference
CNTs / Nafion<br>Su-CNTs / Nafion	+141% more strength	Negligible at low RH Negative at RH > 20%<br>Cont. Angle: N/A	1.3 × 10^{-6} S/cm	[2]
Graphene / Nafion	+131% more strength (dry)<br>+135% more strength (wet)	+282% water uptake<br>Cont. Angle: N/A	No current flowing<br>Polarization did not occur	[3]
Su-CNTs / Nafion	Reduced swelling	-123% water uptake<br>-121% swelling ratio<br>Cont. Angle: N/A	Value not reported	[4]
Graphene / Ag / Nafion	N/A	+257% water uptake<br>Cont. Angle: N/A	Value not reported	[5]
GO / Nafion	+259% more strain<br>+145% more strength	+122% swelling ratio<br>Cont. Angle of 76o	2.00 × 10−4 S/cm	Our work

Bibliographic References

Table S1. Mechanical, hydrophilicity and electrical insulation comparison between nanocarbon reinforcement agents used in Nafion composites for PEMWE applications.

[1] M. N. Silberstein, “Mechanics of Proton Exchange Membranes: Time, Temperature and Hydration dependence of the Stress-Strain behavior of Persulfonated Polytetrafluorethylene,” Massachusetts Institute of Technology, 2008.

[2] C. Yin, J. Li, J. Zhou, H. Zhang, P. Fang, and C. He, “Enhancement in Proton Conductivity and Thermal Stability in Nafion Membranes Induced by Incorporation of Sulfonated Carbon Nanotubes,” ACS Appl. Mater. Interfaces, vol. 10, 2018, doi: 10.1021/acsami.8b01513.

[3] D. Ion-Ebrasu et al., “Graphene modified fluorinated cation-exchange membranes for proton exchange membrane water electrolysis,” Int. J. Hydrogen Energy, vol. 44, no. 21, pp. 10190–10196, 2019, doi: https://doi.org/10.1016/j.ijhydene.2019.02.148.

[4] L. Qian et al., “Magnetic aligned sulfonated carbon nanotube/Nafion composite membranes with anisotropic mechanical and proton conductive properties,” J. Mater. Sci., vol. 56, no. 11, pp. 6764–6779, Apr. 2021, doi: 10.1007/s10853-020-05678-0.

[5] C. S. Yesaswi, S. K. Sahu, and P. S. R. Sreekanth, “Experimental Investigation of Electro-Mechanical Behavior of Silver-Coated Teflon Fabric-Reinforced Nafion Ionic Polymer Metal Composite with Carbon Nanotubes and Graphene Nanoparticles,” Polymers (Basel)., vol. 14, no. 24, 2022, doi: 10.3390/polym14245497.
